# International Engagement Is Critical to Fighting Epidemics

**DOI:** 10.1089/hs.2016.0098

**Published:** 2017-02-01

**Authors:** Jennifer B. Nuzzo, Matthew P. Shearer

In the past 15 years, a series of infectious disease emergencies—the anthrax attacks in 2001, the rapid global spread of severe acute respiratory syndrome (SARS) in 2003, the 2009 influenza A (H1N1) pandemic, the emergence and international spread of the Middle East respiratory syndrome coronavirus (MERS-CoV), the largest Ebola epidemic on record, and the emergence and spread of Zika virus—have increased global political concerns about emerging infectious disease threats and deliberate epidemics. Among these events, Ebola and Zika serve as stark reminders that, if left unchecked, infectious disease outbreaks that originate in one country can produce profound international human, political, and economic consequences. Limited public health and healthcare infrastructure in West Africa quickly enabled Ebola to rapidly spread to multiple countries, resulting in unprecedented levels of illness and death. The suffering caused by the epidemic led to social unrest and economic distress that threatened to undermine decades of US investment aimed at bringing political stability to the region. To help end the epidemic, the US Congress appropriated more than $5 billion in emergency funds.

Although it was first identified in Uganda in 1947, global concerns about Zika virus accelerated last year when Brazil announced an association between Zika virus infection and a birth defect known as microcephaly. To date, Zika infections have been reported by 69 countries, including the United States, which has identified more than 37,000 cases (including territories).^1^ The costs associated with this crisis have been staggering. The World Bank estimates that the short-term economic impact of Zika in Latin America and the Caribbean will be upwards of $3.5 billion.^2^ Though estimates of the short- and long-term economic impacts on the United States are not available, the CDC has estimated that it may cost up to $10 million to provide long-term care for each child who is born with Zika-associated birth defects.^3^

What we have learned from such events is that there are consequences in the United States and other countries when the nations that first encounter an outbreak lack adequate public health infrastructure to detect and respond to acute infectious disease events. When countries are unable to recognize and contain outbreaks, it increases the risk that the diseases will quickly move beyond their borders. The more a disease spreads beyond its source, the harder and more costly it is to contain. This holds true for both natural epidemics and biological attacks.

The increasing recognition that any significant infectious disease outbreak occurring abroad may pose a threat to America has prompted the United States to work with other nations to bolster their response to public health emergencies as a means of protecting national security. Over the past 15 years, US leadership has been important in ensuring the success of a number of international initiatives aimed at improving global health security, and, moving forward, the United States should continue to build on progress made to date. Specifically, it is in the best interest of the United States to pursue as a matter of priority the international initiatives discussed below.

## Recommendations

➢ **Maintain momentum for the International Health Regulations and the Global Health Security Agenda.**

Following global missteps in controlling the spread of the SARS virus in 2003, the 2005 World Health Assembly adopted revisions to the International Health Regulations (IHR) with the aim of improving national and international efforts to detect and respond to events like SARS and other public health emergencies of international concern. The revised IHR outline specific minimum core public health capacities and implementation processes that nations need to adequately address acute public health threats. Although 194 states parties signed onto this legally binding treaty, progress toward implementation of the IHR has been very slow. The initial target for full IHR implementation was 2012; however, even by 2014, only 33% of nations indicated that they had successfully implemented these minimum requirements.^4^

To jumpstart stalled progress toward implementation of the IHR, the Global Health Security Agenda (GHSA) was established in 2014 with the goal of increasing national government support for IHR implementation and fostering a multilateral and multisectoral approach to developing health security capacity. Currently consisting of more than 50 countries, and with support from intergovernmental organizations (IGOs) and nongovernmental organizations (NGOs), the GHSA provides support for nations to assess their existing health security capabilities, identify gaps, and formulate plans to fully implement the IHR to ensure domestic and international capacity to detect, prevent, and respond to health security threats. One of the driving principles of the GHSA is building interagency coordination at the national level, and particularly improving the links between health and security authorities, to facilitate information sharing and improve response to a wide range of biological events, including the deliberate use of dangerous pathogens.

US leadership through the GHSA has breathed new life into the IHR, drawing much-needed awareness from national governments and providing a multilateral mechanism to provide support to low-resource nations. In 2015, the United States committed to contribute more than $1 billion to the GHSA—with more than half of those resources designated for African nations. Other G-7 nations followed the United States's example and pledged contributions to provide support to a total of more than 60 countries.^5^ The United States has also worked with GHSA partner countries and the WHO to develop a tool for conducting external assessments of countries' baseline health security capacity. The WHO's IHR Joint External Evaluation (JEE) tool builds on the GHSA's focus areas to provide a standard metric by which countries can assess their current baseline capacities and measure future progress toward full development of IHR capabilities.^6^

The WHO currently has a list of dozens of nations who have volunteered to complete formal JEEs—a welcome development. After more than a decade of stalled progress on IHR implementation, it is encouraging to see a new wave of enthusiasm for global health security. To sustain and build on this momentum, the United States must continue to work with the WHO and other GHSA partners to assist countries in assessing their health security capacity and making the necessary improvements to ensure that epidemics no longer pose significant threats to society. This will require continued financial and technical support from the United States for the GHSA and the WHO. US leadership on both of these fronts has been essential to building momentum for the GHSA and the IHR, and it will be vital to expanding the success of this program to bolster both global and domestic health security.

➢ **Promote evidence-based plans for limiting international spread of disease.**

The absence of evidence-based plans—both in the United States and abroad—for limiting the spread of disease continues to hinder global response to outbreaks. During the Ebola epidemic in West Africa, there were contentious debates in the United States over whether and how to implement disease control measures at national borders and points of entry. After the importation of a single case of Ebola by a traveler from West Africa, political leaders called for a ban on commercial travel from West Africa and mandatory quarantine for all healthcare workers returning from the region. Health authorities, including the WHO and CDC, deemed that travel bans would cause more harm than good and cautioned strongly against them. Additionally, some states defied CDC recommendations regarding returning traveler assessment and monitoring and implemented mandatory quarantines and movement restrictions on travelers from West Africa.

National leaders should take a strong stand against the imposition of non–evidence-based measures to control the spread of disease. Past experience has shown that when countries employ public health measures such as quarantine and travel restrictions when they are not likely to be effective, they may exacerbate the toll of the outbreak. For example, during the West Africa Ebola epidemic, there were many calls for the United States to ban travel from affected countries—including some from high-ranking members of Congress.^7^ As commercial airlines began voluntarily canceling flights to and from the affected countries, reports surfaced of medical personnel and supplies experiencing inordinate delays getting to West Africa. At a time when clinical care and supplies—particularly personal protective equipment—were in critically low supply, these delays likely contributed to countless additional deaths and actually caused further spread of the disease rather than mitigating it.^8,9^

In future outbreaks, the United States would be in a better position to deter other countries from taking harmful actions—such as the unwarranted detainment of American airline passengers and other unnecessary restrictions on travel and trade—if we openly commit to employ in the United States only those measures that are based on scientific evidence. Measures such as quarantine, point-of-entry screening, and traveler monitoring should be implemented only when there is reasonable evidence that they will be effective. Additionally, the United States should lead by example by developing domestic plans for containing the spread of disease that have the strong backing of scientific evidence.

Countries that restrict travel during infectious disease events without scientific justification do so without the backing of international law. The core goal of the IHR—the legal framework that articulates how nations, including the United States, should respond to international disease threats—is to “prevent, protect against, control and provide a public health response to the international spread of disease in ways that are commensurate with and restricted to public health risks, and which avoid unnecessary interference with international traffic and trade.”^10^ The IHR explicitly discourage the use of public health measures for which scientific evidence is lacking.

➢ **Seek international consensus on data sharing.**

Though recent changes to the IHR have improved the timeliness with which countries report potential public health emergencies of international concern to WHO, there continues to be a lack of consensus regarding whether and how countries should collect and share other data that are needed to inform management of outbreaks. Recently, WHO has worked with countries to address some of these concerns by developing the Pandemic Influenza Plan (PIP) to guide the sharing of influenza specimens and isolates, which are needed to understand the changing epidemiology of influenza outbreaks and to inform vaccine and diagnostic development efforts. However, the plan does not establish a global consensus for how countries, NGOs, IGOs, academics, and private companies (eg, pharmaceutical and biotech sectors) should collect and share surveillance information and collaborate on research in the midst of acute infectious disease emergencies. Sharing data and samples is essential because the availability of accurate, reliable, timely data drives the ability to investigate and respond to infectious disease events and is vital to informing public health control measures and for supporting efforts to develop and test new pharmaceuticals. Considering rapid advancements in public health informatics and biomedical research, the United States should work with WHO and its international partners to develop and adopt an international consensus framework of best practices to guide collaborative research during outbreaks, regardless of the pathogen.

## Conclusion

A number of recent events have highlighted the threat of the continued emergence and spread of epidemic diseases. While the vast majority of these threats begin abroad, increased globalization has ensured that these diseases are merely a flight away. The United States, through international mechanisms such as the GHSA, can provide support for other nations to implement surveillance, preparedness, and response capacity for natural, accidental, and deliberate threats. With these systems in place globally, these types of events can be identified rapidly and contained using national or regional response assets. International cooperation to share data and biological material can support rapid investigations and pharmaceutical development to limit the global impact of emerging diseases, and international standards for evidence-based public health and medical interventions will ensure that responses are managed effectively with minimal impact on international trade, travel, and the US and global economies. International engagement is vital to addressing disease threats before they ever reach our borders as well as mitigating their impact on the global economy and health.

## References

[1] Zika virus: case counts in the US. Centers for Disease Control and Prevention website. Updated 11 23, 2016 http://www.cdc.gov/zika/geo/united-states.html Accessed 1130, 2016

[2] World Bank Group. The short-term economic costs of Zika in Latin America and the Caribbean (LCR). 2 18, 2016 http://pubdocs.worldbank.org/en/410321455758564708/The-short-term-economic-costs-of-Zika-in-LCR-final-doc-autores-feb-18.pdf Accessed 1130, 2016

[3] Frieden T, Fauci AS CDC and NIH officials: how not to fight the Zika virus. Washington Post 8 31, 2016 https://www.washingtonpost.com/opinions/cdc-and-niaid-officials-congress-is-showing-how-not-to-fight-the-zika-virus/2016/08/31/c2efc146-6f7f-11e6-8365-b19e428a975e_story.html Accessed 1130, 2016

[4] World Health Assembly Review Committee on Second Extensions for Establishing National Public Health Capacities and on IHR Implementation. Implementation of the International Health Regulations (2005). 1 16, 2015 http://www.who.int/ihr/B136_22Add1-en_IHR_RC_Second_extensions.pdf Accessed 1130, 2016

[5] Fact sheet: the Global Health Security Agenda [press release]. Washington, DC: The White House, Office of the Press Secretary; 7 28, 2015 https://www.whitehouse.gov/the-press-office/2015/11/16/fact-sheet-us-commitment-global-health-security-agenda Accessed 1130, 2016

[6] World Health Organization. Joint External Evaluation Tool: International Health Regulations (2005). Geneva: World Health Organization; 2016 http://apps.who.int/iris/bitstream/10665/204368/1/9789241510172_eng.pdf?ua=1 Accessed 1130, 2016

[7] Zavis A Ebola crisis: should the U.S. impose an air travel ban or not? Los Angeles Times 10 16, 2014 http://www.latimes.com/world/africa/la-fg-ebola-air-travel-20141016-story.html Accessed 121, 2016

[8] Northam J Ebola supply shipments delayed by transportation issues [transcript]. Morning Edition National Public Radio. 9 8, 2014 http://www.npr.org/2014/09/08/346735497/ebola-supply-shipments-delayed-by-transportation-issues Accessed 121, 2016

[9] Frieden T CDC Chief: why I don't support a travel ban to combat Ebola outbreak. Fox News 10 9, 2014 https://blogs.cdc.gov/global/2014/10/13/cdc-director-why-i-dont-support-a-travel-ban-to-combat-ebola-outbreak/ Accessed 121, 2016

[10] World Health Organization. International Health Regulations (2005). 3rd ed. Geneva: World Health Organization; 2005 http://apps.who.int/iris/bitstream/10665/246107/1/9789241580496-eng.pdf?ua=1 Accessed 1130, 2016

